# Comparative dosimetric analysis of volumetric modulated arc therapy based craniospinal irradiation plans between Halcyon ring gantry and TrueBeam C-arm linear accelerator

**DOI:** 10.1038/s41598-023-30429-x

**Published:** 2023-03-01

**Authors:** Biplab Sarkar, Subhra Snigdha Biswal, Tanweer Shahid, Tanmoy Ghosh, Jibak Bhattacharya, Arundhati De, Mukti Mukherjee, Tharmarnadar Ganesh, Luca Cozzi

**Affiliations:** 1 Department of Radiation Oncology, Apollo Multispeciality Hospitals, Kolkata, India; 2 Manipal Hospitals, Dwarka, New Delhi India; 3 Radiotherapy and Radiosurgery Department, Humanitas Research Hospital and Cancer Center, Via Manzoni 56, 20089 Milan, Rozzano Italy; 4 Department of Biomedical Sciences, Humanitas University, Via Rita Levi Montalcini 4, Pieve Emanuele, 20090 Milan, Italy; 5 Varian Medical Systems, Palo Alto, USA

**Keywords:** Cancer, Radiotherapy, Cancer therapy, CNS cancer

## Abstract

This study evaluates the volumetric modulated arc therapy (VMAT) dosimetric comparison between Halcyon ring gantry and TrueBeam c-arm linear accelerators for craniospinal irradiation (CSI) of the neuro-axis. 25 patients, who received treatment for medulloblastoma and primitive neuro-ectodermal tumors between 2018 and 2021, were planned for VMAT in True Beam (TB), and Halcyon (HAL) linear accelerators using 6 MV unflattened (FFF) photon beams (HALFFF and TBFFF). Dose-volume statistics for the target and organs at risk (OARs) and the total number of monitoring units (MUs) in the treatment plans were compared which included dose received by 95% PTV volume (V95%), volume receiving ≥ 107% dose, homogeneity index (HI), conformity index (PI), MU and dose spillage (D10%, D30%, D50%, D70%, D90%). In all 26 OARs were considered of which five were serial and the remaining were parallel structures. For the former, the dose received by 0.2 cm^3^, volume = D0.2 cm^3^) were evaluated and for the latter mean dose were evaluated. Both arms were statistically compared with paired sample t-test with a significant value of ≤ 0.05. 11 patients received treatment with the Halcyon and the rest 14 in the TrueBeam C-arm linear accelerator. Patients in the low- and intermediate-risk category (n = 13) received 23.4 Gy in 13 fractions. The remaining patients were in the high-risk category and received 35 Gy in 21 fractions or 36 Gy in 20 fractions. For HALFFF and TBFFF, PTVV95% were 97.5 ± 0.8% and 97.4 ± 0.9% respectively (p = 0.371) while the V107% were 0.6 ± 0.4% and 0.5 ± 0.5 respectively (p = 0.504). However, the number of monitoring units showed statistical significance (p < 0.001) with values of 1331.9 ± 243.4 MU and 1089 ± 206.7 MU respectively for the HAL and TB plans. The differences in spillage dose were also statistically significant, favouring HAL plans at D30% (p = 0.002), D50% (p < 0.001), D70% (p = 0.039), and D90% (p = 0.01) level except for D10% (p = 0.090). Conformity index also showed statistical significance with PI_HAL = 0.9 ± 0.02 and PI_TB = 0.89 ± 0.03 (p = 0.029). For 10 of the 21 parallel structures, the mean dose differences were statistically significant in favouring of HAL plans. Halcyon based VMAT CSI plans are dosimetrically superior in terms of organ dose, especially for the large organs, and offer lower spillage doses than the TrueBeam plans. Plans generated by both linear accelerators are suitable for the patients’ treatments.

## Introduction

Medulloblastoma and primitive neuro-ectodermal tumors (PNET) are among the few most common paediatric tumours^1^. Post-surgery management of medulloblastoma and a few variants of PNET (like ependymoma) requires radiotherapy to the complete craniospinal axis (CSI) along with the chemotherapy in a neoadjuvant setting followed by maintenance chemotherapy^1^. Radiotherapy planning of the craniospinal axis remains an intricate task because of the hurdles involved in creating a uniform dose to the brain and the spinal axis, avoiding hot and cold spots at the field junctions. Challenges involved in craniospinal irradiation planning include over- or under-dosing at the brain-spine and spine-spine field junctions. These over-and under-dose regions arise due to the overlay of or the gap between the adjacent treatment fields. Radiotherapy treatment plans for craniospinal irradiation have evolved over a period of time from simple, conventional simulator-based junction shift technique to CT-based three-dimensional conformal radiotherapy (3D-CRT) with moving junctions, followed by junction overlapping intensity modulated radiotherapy (IMRT) and finally volumetric modulated arc therapy (VMAT) based low-gradient junction techniques^2–8^. The age group of the patients is either adolescent or childhood, and five-year recurrence-free survival rates are above 80% in children with localised disease and close to 70% in patients with high-risk features. One of the most relevant long-term complications of radiotherapy in these patients is neurocognitive dysfunction, growth retardation/anomalies, and quality of life^9–11^. Modern radiotherapy techniques have made it possible to sculpt the dose for better uniformity over the craniospinal axis, reducing the possibility of radiation comorbidities in the future.

Presently, the VMAT-based low-gradient junction CSI technique is the most preferred choice for CSI treatment, as unlike 3D-CRT, it does not require a junction shift after certain number of treatment fractions^6–8^. Minor systematic shifts in the patient’s position do not affect the junction doses and dose homogeneity^7^. Further, it produces a more uniform dose distribution throughout the target volume than the 3D-CRT technique. First described by Fogliata et al., in 2011, different properties of the VMAT-based CSI technique were subsequently investigated by several researchers and the technique is currently well adapted in clinical practice.^6–8,12–16^. These investigations were limited to the c-arm linear accelerator or helical Tomotherapy (Accuray Inc., Sunnyvale, CA) treatment delivery systems^5–8,12–17^. Recently, Varian Medical Systems (Palo Alto, CA) has introduced a ring-gantry type treatment delivery system called Halcyon. There are several differences between the Halcyon and TomoTherapy, including the delivery parameters and multileaf collimator (MLC). The most notable differences are: (i) Halcyon delivers the therapy volumetrically and differs from the sliced delivery of helical TomoTherapy, (ii) during radiation delivery, the table remains static for Halcyon but it moves for TomoTherapy; and (iii) Halcyon uses two banks of strip MLCs while Tomotherapy uses a binary MLC.

Recently, one of the two C-arm linear accelerators in our center was replaced by a Halcyon (model E) linear accelerator in August 2020. After initial internal evaluation of treatment planning and quality assurance tests, the first CSI patient was treated in Halcyon in November 2020. Until August 2022, we have treated 11 CSI patients^18^. VMAT-based CSI treatment plans using Halcyon were clinically acceptable in all dosimetric parameters. Nonetheless, we designed a large scale comparative dosimetric study comprising 25 patients to find the best possible treatment plan between Halcyon (HAL) and TrueBeam (TB) (both from Varian Medical Systems, Palo Alto, CA) linear accelerators using 6 MV flattening filter free (FFF) beams. As a corollary, and for the completeness of the study, we also compared the dosimetric parameters of the plans using the 6 MV flattened beam of the TB linear accelerator (TBFF).

## Materials and methods

### Characteristics of the linear accelerators

The Halcyon model E, with the source-to-isocentre distance of 100 cm, has no backup jaws, and is equipped with two staggered stacks of 1-cm width MLC with an effective resolution of 5 mm, defining a largest field opening of 28 × 28 cm^2^ and one flattening filter-free x-ray beam of 6 MV with a maximum dose rate of 800 MU/min^19^. The TrueBeam linear accelerator used in this study has a 40 × 40 cm^2^ maximum field size defined by MLC and (or) backup jaws. It has a 120 leaf millennium MLC, consisting of two opposing leaf banks with leaves that traverse along the X-axis. For the central 20 cm of the MLC, each leaf has a width of 5 mm at the isocenter, whereas for the peripheral 10 cm on either side, the leaf width is 10 mm. Both linear accelerators are calibrated to deliver 1 cGy/MU at the depth of dose maximum (dmax) for a source-to-surface distance of 100 cm.

### Patient selection criteria

The twenty-five patients included in this study were treated between September 2018 and September 2021. Of these, 11 patients received treatment in the newly installed Halcyon and the remaining 14 patients were treated in the TrueBeam linear accelerator. For dosimetric comparison between the two linear accelerators, a comparative plan was retrospectively created for each patient in the alternate linear accelerator, leading to 14 and 11 non-treatment plans of HALFFF and TBFFF, respectively. Furthermore, all 25 plans were compared dosimetrically for target volume coverage, hot volume (% target volume receiving more than 107% of the prescription dose), organ at risk (OAR) doses, dose to normal tissue at different levels, and number of monitor units.

### Simulation and contouring

All patients were simulated in a head-first, supine position, immobilised with five-clamp thermoplastic covering the brain to chest level. If the patient was non-cooperative and no sedition was used, an additional two-clamp thermoplastic was used to immobilise the abdomen pelvis region^7^. Anesthesia was used if needed. All patients were simulated in a Brilliance Big Bore CT scanner (Philips, Eindhoven, The Netherlands) with 3-mm uniform slice thickness from brain to mid-thigh, with the first marker in the brain and second marker at the abdomen level to keep the patient straight during the simulation. CT Images were transferred to the SomaVision (Varian Medical Systems, Palo Alto, CA) contouring station and co-registered with three-dimensional (3D) T1-contrast, T2-flair magnetic resonance images (MRI).

The gross tumour volumes (GTV) of the brain and the spine were delineated as follows: the cranial contouring included the whole brain and up to the junction of the cervical vertebrae C5 and C6. The superior end of the spinal cord starts from the end of brain GTV and goes up to the inferior end of the thecal sac, as seen on the sagittal view of the MRI. The planning target volume (PTV) for the brain was generated by applying a 3 mm margin on the GTV. For the spinal cord, the PTV was generated using a 7 mm margin over GTV^7^. The brain and spinal PTVs were summed to generate a single PTV for the plan optimisation. To standardise the contouring of organs at risk for all patients, a predefined structure template consisting of bladder, bowel, brain stem, chiasm, cochlea (bilateral), duodenum, esophagus, eyes (bilateral), thyroid gland, heart, humerus head (bilateral), kidneys (bilateral), lacrimal gland (bilateral), larynx, lens (bilateral), lung (bilateral), mandible, optic nerve (bilateral), oral cavity, ovary (bilateral for female patients), parotid (bilateral), pituitary gland, rectum, stomach, and submandibular glands (bilateral) was used.

### Treatment Planning

**Figure 1:**
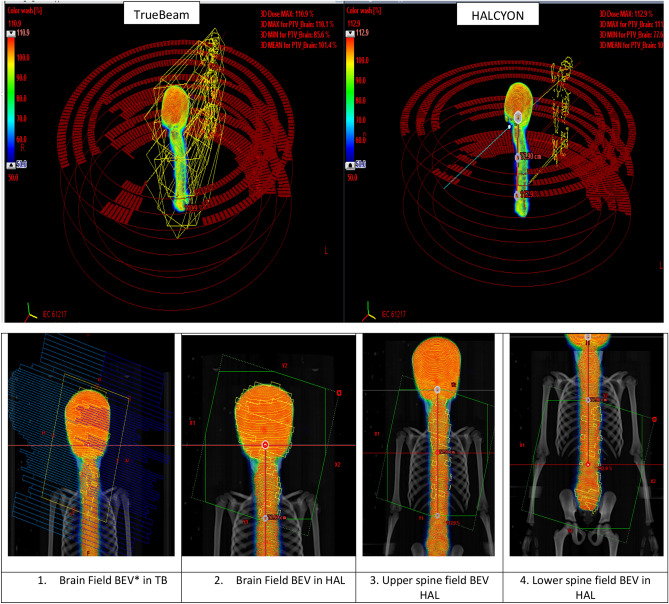
Upper Panel: Arc arrangement and dose distribution for Halcyon and TrueBeam linear accelerators. Lower Panel: (left to right) *Beams Eye View (BEV) for TrueBeam-Brain field, HAL BEV (1) brain field, (2) upper spine, and (3) lower spine field. Field overlap between the anatomical region is visible between image 2–4.

All treatment plans were planned with 6 MV FFF photon beams at the dose rate of 800 MU/min for HAL an 1400 MU/min for TB in the Eclipse V15.6 treatment planning system (TPS). For dosimetric comparison, TBFF plans were created from the TBFFF plan without changing any optimisation parameter. For both HAL and TB, the same optimisation engine (photon optimiser) and the same dose calculation algorithm (Analytical Anisotropic Algorithm (AAA)) were used. The number of isocentres and their placement were decided based on an adjacent field overlap of 10 cm^7^. Craniocaudal PTV length was divided equally in an even number of sectors. For plans with two isocentres, the total PTV length was divided into four sectors and the first and the second isocentre were placed at the first and third interval. For longer PTVs, which cannot be covered using two isocentres, an additional isocentre was placed using the same strategy. Only longitudinal shift was allowed between the first and subsequent isocentres. The number of isocentres differed between HAL and TB plans due to differences in the field size. Further details on the choice of number of isocentres and isocentre placement strategy can be found in an earlier study^7^. CSI planning and the resultant dosimetric metrics are highly dependent on the chosen arc length. Historically, all Varian users used a full or very large arc length for the spine fields^6,8^. However, we adopted the planning strategy from Sarkar et al., where the spinal fields were treated with a partial posterior arc instead of the commonly used full arc technique in the Eclipse TPS^6,8,20^. Spine arc length was increased to 140° instead of the original 100°^14,20^. Early studies established that full/large arc angle was dosimetrically inferior and associated with increased spillage dose and OAR doses, without any improvement in conformity or homogeneity^7^. The 140° posterior arc length was sufficient for spinal PTV. Two full 360° arcs with avoidance sectors (250°-0°-110° in the clockwise direction and 110°-0°-250° in the anti-clockwise direction) were used instead of two ± 70° shorter arcs about 180° for the spinal PTV. For the brain isocentre, full arcs were used. Figure 1 represents the arcs arrangement and dose distribution for HAL (left) and TB (right) plans. For all Halcyon plans, we used three arcs for each isocentre with collimator angles of 285°, 345° and 45°, respectively. All optimisation parameters were kept the same between all arms, with only few slight modifications done as per specific plans' requirements. The HALFFF and TBFFF and TBFF plans were evaluated for the target dose coverage, hot volume, MU, Paddick conformity index (PI = $\frac{{V}_{Rx}^{2}}{TV\times {V}_{RI}}$) where TV is the volume of the target, V_RX_ is the volume of target covered by prescribed isodose (95%) and V_RI_ is the volume of tissue receiving prescription dose (95%)^21^. RTOG Homogeneity index (HI = D_≥95% (within PTV)_/D_≥5% (within PTV)_)^22^. Different levels of spillage dose by calculating “body volume” receiving 10% (D10%), 30% (D30%), 50% (D50%), 70% (D70%) and 90% (D90%) of the prescription dose. Pairwise statistical analysis was carried out using paired sample t-test, while a combined three-arm statistics was evaluated using one way ANOVA. Statistical significance was defined at p < 0.05.

### Ethical approval

Apollo Multispeciality Hospital, Kolkata Ethical committee approved the study. All methods used in this article were carried out in accordance with relevant guidelines and regulations of the Apollo Multispeciality Hospital, Kolkata. All experimental protocols were approved by Apollo Multispeciality Hospital, Kolkata Ethical committee. All participants and/or their legal guardian provided the informed consent to participate in this study.

## Results

For the eleven patients treated in the Halcyon unit, in addition to the HALFFF plans used in the treatment, eleven non-treatment TBFFF plans were created. Similarly, for the fifteen patients treated in the TrueBeam unit, in addition to the TBFFF plans used in the treatment, fifteen non-treatment HALFFF plans were created. Additionally, 25 TBFF non-treatment plans were generated for comparison purposes. The average age of the patients was 12.3 ± 7.8 years (female 7 and male 19). The diagnosis and disease characteristics, PTV volume, and length are presented in Table 1.

**Table 1:** Diagnosis and disease characterises.

Age (year)	< 4.5|13.0 ± 8.5|41 >
Sex	
M	20
F	5
Diagenesis	
Medulloblastoma	17
PNET	6
Ependymoma	2
PTV	
Length (cm)	< 43|62.3 ± 10.6|76.5 >
Volume (cm^3^)	< 929.5|2015.9 ± 337.3|2642.9 >
Prescription dose	
Average dose (Gy)	< 23.4|29.3 ± 6.2|36 >
High risk	36 Gy in 20 Fractions or (8) 35 Gy in 21 Fractions (4)
Intermediate and low risk	23.4 Gy in 13 Fractions (13)

Institutional plan acceptability criteria were 95% target volume receive at least 95% of the prescribed dose and less than 2% volume receives ≤ 107%. Both conditions were satisfied for all the 75 treatment plans generated on HALFFF, TBFFF, and TBFF beam energies. Average dose (< *Lower limit|Mean* ± *Standard Deviation|Upper Limit* >), received by 95% of the PTV volume (D95%PTV) for HALFFF, TBFFF and TBFF plans were < 95.6|97.5 ± 0.8|99.2 > %, < 95.3|97.4 ± 0.9|99.1 > %, and < 95.1|97.7 ± 1.03|98.2 > % respectively and the difference is not statistically significant (p = 0.232:ANOVA). Volume receiving ≥ 107% dose, in same sequence, were < 0|0.6 ± 0.4|1.5 > %, < 0.0|0.5 ± 0.5|1.8 > %, and < 1.1|1.5 ± 0.4|2.5 > with a statistically insignificant difference (p = 0.312). MUs for HALFFF and TBFFF plans were < 1024.4|1331.9 ± 243.4|1983.4 > and < 730.6|1089 ± 206.7|1665.3 > respectively with a mean difference of < 64.4|242.9 ± 133.7|444 > MUs and found statistically significant (p < 0.001). TBFF mean MUs was < 887|1260 ± 265.0|1858 > and mean difference with HALFFF MU was < 128|129.1 ± 157.3|256.4 > and statistically significant p < 0.001.

**Figure 2:**
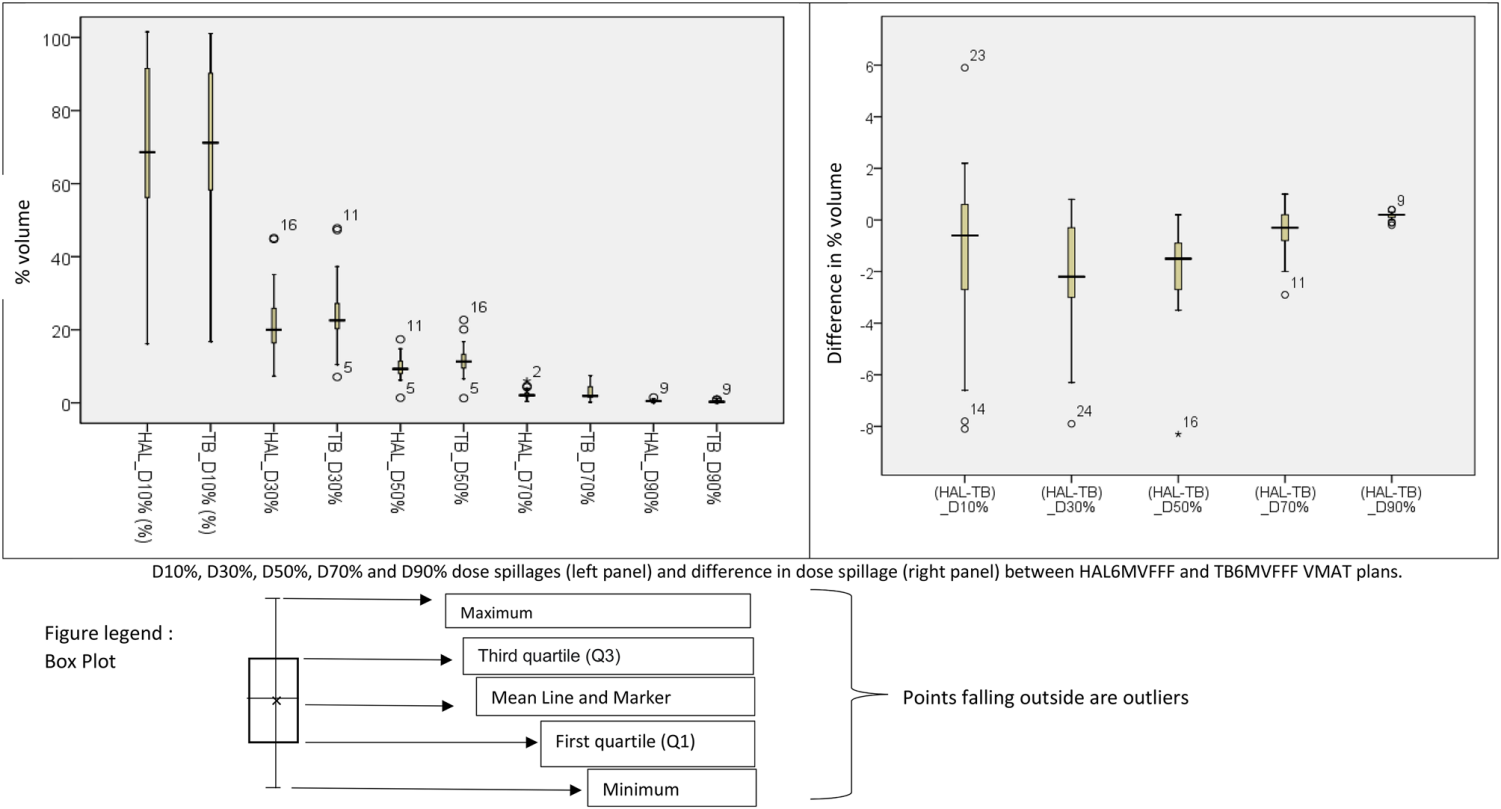
D10%, D30%, D50%, D70% and D90% dose spillages (left panel) and difference in dose spillage (right panel) between HAL and TB plans.

D10%, D30%, D50%, D70% and D90% dose spillages (left panel) and difference in dose spillage (right panel) in two competing arms presented in Fig. 2. The number of MUs in Halcyon plans was, on an average, higher by 300 MUs than that in TrueBeam plans. HAL plans showed lesser dose spillage to the body. Statistical analysis of each of the dose levels shows a significant difference at D30% (p = 0.002), D50% (p < 0.001), D70% (p = 0.04), and D90% (p = 0.01) level except at the lowest dose (highest volume) level of D10% (p = 0.09). PI for HALFFF, TBFFF, and TBFF were 0.90 ± 0.02, 0.89 ± 0.03, and 0.87 ± 0.05 respectively, HI for HALFFF, TBFFF, and TBFF were 1.08 ± 0.02, 1.08 ± 0.01, and 1.07 ± 0.02 respectively. The difference between HALFFF and TBFFF PI (p = 0.03) was statistically significant, and HI (p = 0.7) was statistically neutral. Similar conditions were obtained between HALFFF and TBFF, with PI difference significant at p = 0.04 and HI difference insignificant at p = 0.5.

A total of 12 patients, who were in the high/intermediate-risk group, received 35 Gy or 36 Gy radiation, while the rest 13 patients received treatment for a 23.4 Gy prescription dose. Table 2 shows the mean relative OAR doses and statistical test results.

**Table 2:** Relative OAR doses between Halcyon FFF and True Beam FFF and True Beam FF arm. Statistical significance difference at p ≤ 0.05 using paired sample t-test.

	HALFFF (%)	TBFFF (%)	TBFF (%)	p (HALFF Vs.TBFFF)	p (HALFF Vs.TBFF)	p (TBFFF Vs. TBFF)	(HAL-TB) FFF (%)	(HAL-TB) FF (%)	TB (FFF-FF) (%)
D0.2 cc									
Brainstem	105.0 ± 1.5	105.7 ± 1.9	104.8 ± 1.9	0.33	0.543	0.698	− 0.5 ± 1.8	− 0.3 ± 2.4	0.2 ± 2.5
B/L Cochlea	98.5 ± 7.8	100.6 ± 3.2	100.6 ± 2.9	0.101	0.171	0.51	− 2.1 ± 7.2	− 0.9 ± 4.1	− 0.3 ± 2.9
B/L Lens	23.9 ± 10.9	25.1 ± 13.3	22.7 ± 10.4	0.412	0.315	0.786	− 1.2 ± 9.8	2.1 ± 9.2	7.1 ± 11.4
B/L Opt N	99.1 ± 6.5	97.6 ± 8.8	99.2 ± 6.3	0.076	0.506	0.461	1.4 ± 4.6	− 1.1 ± 7.6	− 0.9 ± 5.9
Opt Chiasma	102.5 ± 2.1	103.5 ± 2.8	101.7 ± 1.9	0.018	0.326	0.13	− 0.8 ± 1.8	0.4 ± 3	0.9 ± 2.7
Mean dose in %									
B/L Eyes	29.2 ± 11.8	31.3 ± 14.0	31.7 ± 11.9	0.015	0.018	0.437	− 2.2 ± 5.9	− 2.2 ± 6.5	− 0.6 ± 7.3
Bladder	4.3 ± 3.8	4.7 ± 3.7	5.2 ± 3.7	0.049	0.091	0.635	− 0.4 ± 1.0	− 0.9 ± 2.6	− 0.3 ± 2.6
Duodenum	29.3 ± 14.9	29.2 ± 14.4	30.2 ± 16.4	0.938	0.856	0.955	0.2 ± 1.5	0.1 ± 6.2	− 0.1 ± 6
Oesophagus	46 ± 14.0	47.6 ± 16.7	48.4 ± 15.8	0.222	0.156	0.226	− 1.6 ± 6.1	− 4.3 ± 6.5	− 2.8 ± 4.2
B/L Femur Head	2.1 ± 2.2	2.6 ± 2.9	3 ± 2.8	0.114	0.083	0.269	− 0.3 ± 1.1	− 0.8 ± 1.2	− 0.4 ± 1.3
Bowel	20.5 ± 5.9	22.5 ± 6.2	25.3 ± 7.4	< 0.001	0.011	0.078	− 2.0 ± 1.9	− 4.7 ± 5	− 2.1 ± 4.3
Heart	16.3 ± 4.9	17.5 ± 5.6	16.5 ± 7.5	0.041	0.954	0.43	− 1.2 ± 2.7	0.3 ± 6.4	1.5 ± 7.5
B/L Kidney	16.5 ± 6.1	17.9 ± 7.5	19.3 ± 6.9	0.031	< 0.001	0.022	− 1.4 ± 3.4	− 2.8 ± 4.5	− 1.4 ± 3.9
Larynx	29.2 ± 5.0	28.2 ± 5.2	28.6 ± 5.9	0.324	0.257	0.768	1.0 ± 5.0	1.4 ± 6.4	0.5 ± 4
Liver	16.4 ± 4.4	17.4 ± 4.7	16.6 ± 4.8	< 0.001	0.035	0.459	− 1.1 ± 1.3	− 0.1 ± 4.5	1.1 ± 4.6
B/L LUNG	20.7 ± 6.9	23 ± 6.9	23.2 ± 6.3	< 0.001	< 0.001	0.544	− 2.3 ± 2.6	− 2.5 ± 3.1	− 0.2 ± 2.2
Mandible	23.2 ± 5.8	23.2 ± 6.1	24.5 ± 9.4	0.983	0.693	0.335	0.0 ± 2.1	− 1.1 ± 3.2	− 1.1 ± 2.1
Pancreas	21.7 ± 7.2	23.4 ± 6.6	24.6 ± 6.7	0.062	0.005	0.018	− 1.6 ± 2.9	− 2.8 ± 3.4	− 1.2 ± 1.9
B/L Parotid	24.8 ± 10.9	25.5 ± 11.8	25.9 ± 11.6	0.493	0.283	0.673	− 0.7 ± 6.6	− 1.1 ± 6.1	− 0.4 ± 5
Rectum	8.8 ± 5.5	10.0 ± 6.3	9.6 ± 7.2	0.02	0.327	0.906	− 1.2 ± 2.3	− 1.2 ± 5.6	0.2 ± 5.7
Stomach	17.0 ± 5.9	18.0 ± 5.5	20.1 ± 7.5	0.114	0.089	0.199	− 1.0 ± 3.2	− 3.5 ± 9.5	− 2.3 ± 8.6
Thyroid	36.6 ± 13.9	36.6 ± 16.3	36.7 ± 15	0.985	0.481	0.676	0.0 ± 4.2	1.2 ± 6.6	1.2 ± 6.6
Total Lung V5Gy (%)	43.6 ± 19.4	53.8 ± 17.4	53.6 ± 20.8	0.023	0.03	0.948	− 10.2 ± 12.6	− 9.9 ± 8.4	0.2 ± 10.8

**Figure 3:**
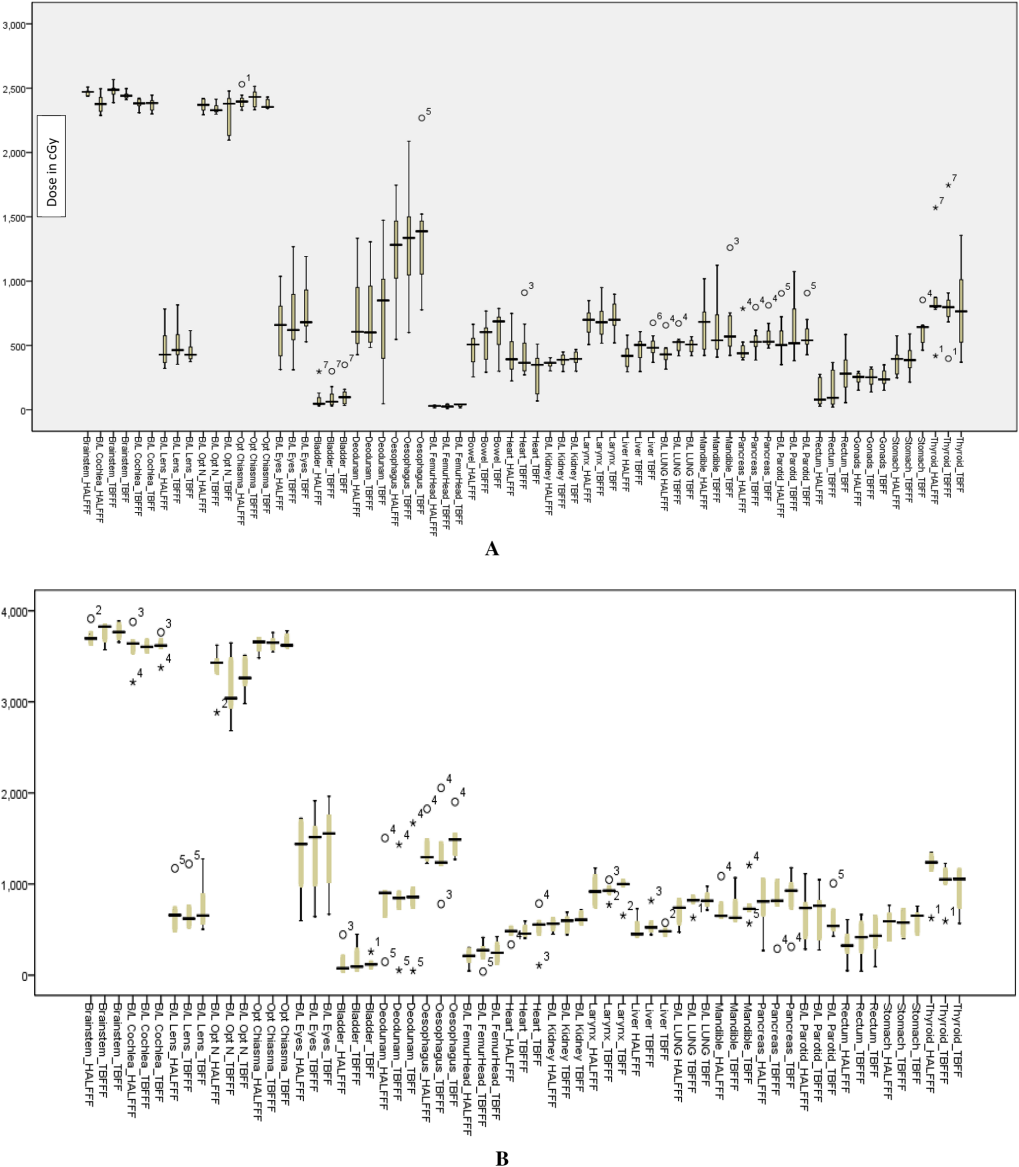
(**A**) Absolute dose to the organ at risk for 23.4 Gy prescription. (Legend meaning: figure-2). (**B**) Absolute dose to the organ at risk for 35 Gy and 36 Gy prescription. (Legend meaning: figure-2).

**Figure 4:**
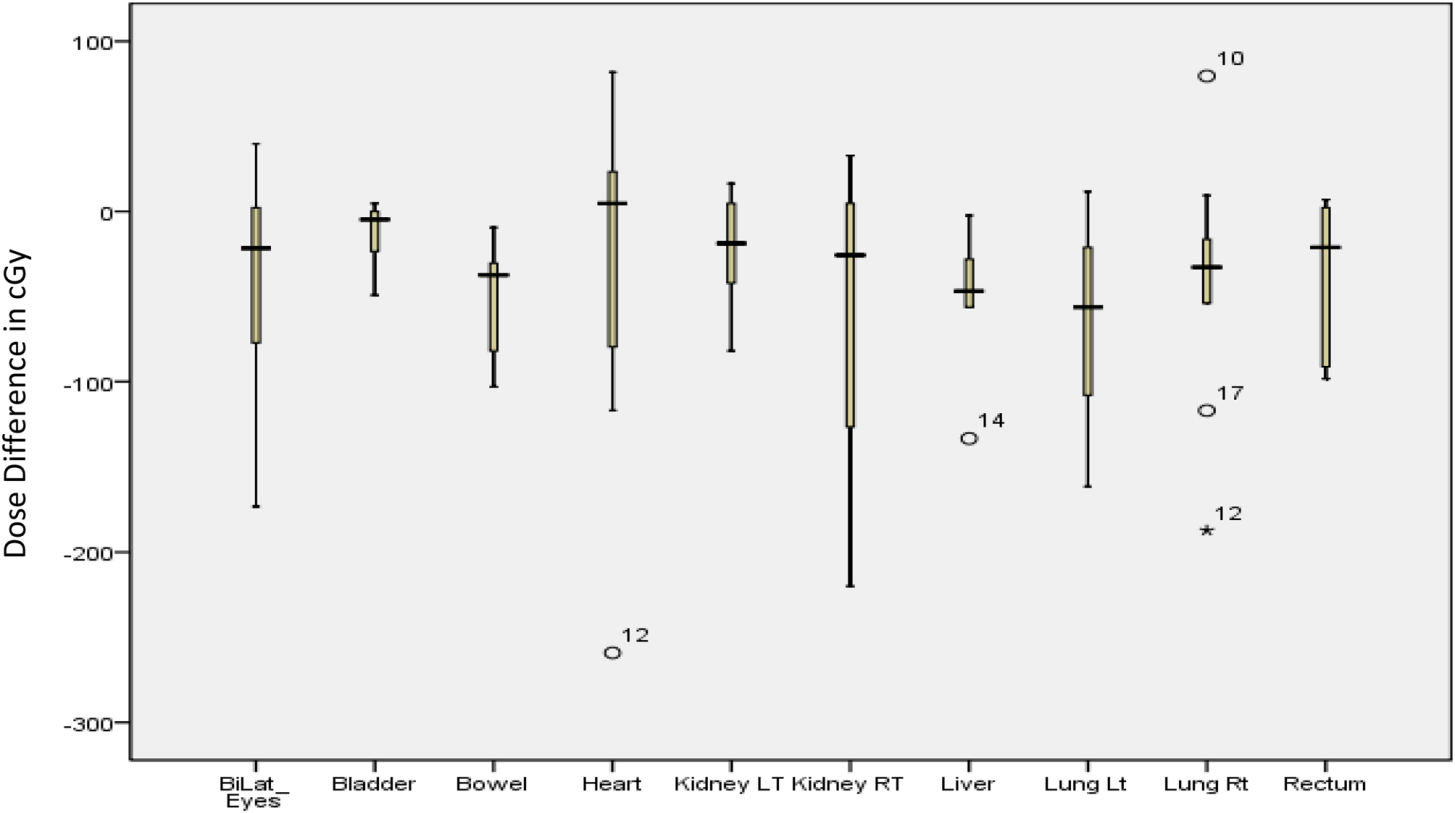
Dose difference for the organ show a statistical difference (p ≤ 0.05) between HAL 6FFF and TBFFF plans.

Of the 26 OARs, 20 OARs showed a lesser dose deposition for Halcyon plans as against the TrueBeam FFF plans, with 10 of them, viz., bladder, bilateral eyes, bowel, bilateral kidneys, bilateral lung, heart, rectum and liver, showing statistically significant difference in their mean doses using paired sample t-test. Similarly, for the optic chiasma, the difference in D0.2cm^3^ was statistically significant between the two types of plans. The difference in total lung V5 Gy (%) was also statistically significant (p = 0.02), favouring the HALFFF plan. A similar result was also found in the comparison between HALFFF and TBFF plans (p = 0.03). Figure 3A,B show the absolute OAR dose comparison between two arms with 23.4 Gy and 36 Gy prescription levels (Supplementary Table S1 presents the same in tabular form). For both the prescriptions, the maximum difference in mean dose between HALFFF and TBFFF was observed for larynx (80.6 ± 178.8 cGy), and the minimum was for mandible (0.8 ± 64.3 cGy). For D0.2cm^3^, the maximum difference was for bilateral optic nerve (109.2 ± 164.2 cGy), and the minimum was for bilateral lens (3.9 ± 110.8 cGy). Only a single organ shows a difference of more than 1 Gy, the bilateral optic nerve; for the rest of the organs, dose difference between HALFFF and TBFFF plans was less than 1 Gy. The statistical difference between HALFFF vs. TBFF was significant for bilateral eyes (p = 0.02), bilateral kidneys (< 0.001), bilateral lung (< 0.001), bowel (p = 0.011), liver (p = 0.04) and pancreas (0.005). Figure 4 shows the box plot for the organs, which shows a statistically significant mean dose distribution for both prescriptions.

## Discussion

Considering the long-term effect of radiotherapy on the long survival of the patients, CSI planning is always considered as one of the most complex planning techniques.^9–11^ The VMAT-based low-gradient junction craniospinal irradiation, using multiple isocentres, is the preferred technique over the junction-based 3D-CRT in terms of better dose uniformity and lesser dose to certain critical organs. Although several authors proposed junction overlap IMRT, it has never been a popular practice in the clinical routine because of the complexity of junction dose optimisation. VMAT/RapidArc based technique for Varian/Eclipse was introduced by Fogliata et al*.* in 2011 and subsequently for Elekta/Monaco by Sarkar et al. in 2014^6,12^. Since the inception of the VMAT-based CSI technique, it has become a popular practice in the routine clinical practice. The modern optimisers perform the junction dose tapering very efficiently without any additional contour, and delivery is also fast. It is now well established for over a decade that the C-arm linear accelerators capable of VMAT/RapidArc can deliver low-gradient junction based CSI efficiently^6–8,12,13,15,16^. This report extends the research for the newly introduced Halcyon-E linear accelerator.

There are several differences between Halcyon (model E) and TrueBeam linear accelerators, which are as follows: (1) gantry speed (2 RPM for HAL and 1 RPM for TB) (2) MLC speed and configuration (5 cm/s for HAL and 2.5 cm/ss for TB) (HAL: double stack, TB: single stack with backup jaws) (3) Halcyon-E has less MLC leakage than TrueBeam (HAL: 0.4% and TB: 0.7%: measured data); (4) Halcyon-E is incapable of delivering non-coplanar beams, whereas TrueBeam is, although, in VMAT-based CSI, non-coplanar beams are not used. As a result, the final point contributes no dosimetric attributes to this study. The dose coverage and hot volume to the craniospinal axis were comparable between two competing arms with no statistical difference. Plan homogeneity was comparable between two-arms. Halcyon shows a statistically improved conformity index and OAR doses for ten organs (bladder, bilateral eyes, bowel, bilateral kidneys, bilateral lung, heart, rectum, and liver, and optic chiasma) and spillage dose. The reason for improved OAR doses is a combination of improved gantry and MLC speed along with less MLC dose spillage compared to TrueBeam. Similar results have been found in an early study by Li et al. for head and neck cancer^23^. The possibility of inert observer variation causing the dosimetric difference is low, as all plans were done by a single user.

Several research groups have compared the performance of the Halcyon linear accelerator to that of the TrueBeam and Novalis Tx linear accelerators for various sites and techniques such as CSI, head neck, liver SBRT, spine SBRT, prostate SBRT, and pelvic node SBRT^14,23–28^. In terms of target coverage, dose conformity, and OAR dose constraints, all authors reported acceptable Halcyon dosimetric plans. Biswal et al. compared craniospinal irradiation in Halcyon and Novalis Tx linear accelerators, which differed significantly in MLC width, beam energy, field size, and gantry speed. They discovered comparable target coverage, statistically improved low dose spillage, and treatment time, favouring Halcyon but with a higher setup margin^10^. Pokhrel et al.^24,25^ demonstrate improved OAR doses and plan quality for prostate, abdominal and pelvic single lymph node irradiation, and lung SBRT. Sarkar et al. contradicted Pokhrel et al. report on lung SBRT; they stated all cases could not have improved dosimetry in Halcyon^25,26^. Visak et al. found that knowledge-based Halcyon planning improved dosimetry over manual Halcyon and non-coplanar TrueBeam lung SBRT plans. Petroccia et al. compared Halcyon spine SBRT to TB plans and found that the former has a slower dose falloff and lower target conformity^27^. According to the literature, Halcyon provided a better overall performance in plan quality and delivery time than comparative C-arm linear accelerators, which supports our result of having a better dosimetric result, with 50% of OARs receiving a statistically significant lower dose than TrueBeam linear accelerators.

Although a difference of mean dose in the two competing arms never exceeded 1 Gy, the inter-quartile range (height of the box in the graph) for these organs is around 1 Gy could be one of the reasons for statistically significant dose distribution. Nonetheless, OAR dose difference may be statistically relevant; it needs to be evaluated for clinical outcome results to establish the superiority of one treatment plan over the other. It will be rather unjustified to claim the superiority of HAL plans over TB plans only based on statistical results. The other influencing factor could be the volume of the organ. Supplementary Fig. S1 shows the different organ volumes in cm^3^. All the large organs, bowel, bladder, bilateral kidneys, bilateral lung, heart, liver, and rectum show a statistically significant dose distribution between HAL and TB plans. However, the exact relationship between the dose difference and the volume of the organ needs further study.

Due to field size limitations, Halcyon required an additional isocentre, hence more MUs. The number of isocentres was a function of three factors: (1) cranio-caudal PTV length, (2) field size in the y direction, and (3) adjacent field overlap of 10 cm. For patients in the paediatric and adolescent age groups (PTV length ≤ 54 cm), TB required 2 isocentres, and HAL required 3 isocentres. With increased PTV length, additional isocenters need to be added. In the current study, 14 cases required HAL-3, TB-2 isocentres, while the remaining 11 cases required HAL-4, TB-3 isocentres. One additional isocentre contributes an average of 300 MUs extra in HAL plans. Nonetheless, with excess MU in Halcyon, the spillage dose D90%, D70%, D50%, and D30% were (statistically) significantly less than the TrueBeam plans. Earlier researchers found improved plan quality in head and neck cancer is contributed by lesser MLC leakage.^23,29^ For HAL, less MLC leakage is further aided by a higher gantry and MLC speed to improve the plan quality and reduce the spillage dose. This is a major finding in our study and may turn out to be dosimetrically significant in par view of AAPM report TG-158, which discusses the out-of-field doses and their contribution to the enhanced risk of second malignancy and cardiac toxicity among the long time cancer survivors^30^. About 10% of the cancer survivors develop second cancer, out of which around 2% is due to early radiotherapy^30,31^. The dose risk (second cancer) relationship is linear in our dose range of interest.

## Conclusion

Clinically and dosimetrically acceptable VMAT-based craniospinal irradiation plans can be generated by the newly introduced Halcyon linear accelerator. In terms of organ at risk doses and low dose spillage, these plans are statistically superior to TrueBeam plans. Nonetheless, the clinically significant advantages of dosimetric superiority must be demonstrated using a systematic outcome result analysis.

## Supplementary Information

Supplementary Figure 1.

Supplementary Table 1.

## Data Availability

The datasets used and analysed during the current study are available from the corresponding author.
