# Supplementary figures and images for: Comparative dosimetric analysis of volumetric modulated arc therapy based craniospinal irradiation plans between Halcyon ring gantry and TrueBeam C-arm linear accelerator
Source: Sci Rep. 2023 Mar 1;13:3430. doi: 10.1038/s41598-023-30429-x (PMC9977918; doi:10.1038/s41598-023-30429-x)

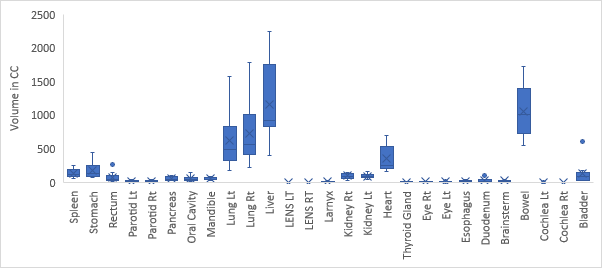


Supplementary Figure-1: Volume of different OAR’s in cm3

Supplement: Supplementary file 1 — Supplementary Figure 1. [file 41598_2023_30429_MOESM1_ESM.docx]
