# Supplementary material for: Comparative dosimetric analysis of volumetric modulated arc therapy based craniospinal irradiation plans between Halcyon ring gantry and TrueBeam C-arm linear accelerator
Source: Sci Rep. 2023 Mar 1;13:3430. doi: 10.1038/s41598-023-30429-x (PMC9977918; doi:10.1038/s41598-023-30429-x)
Supplement: Supplementary file 2 — Supplementary Table 1. [file 41598_2023_30429_MOESM2_ESM.docx]

Supplementary Table-1

|  |  | Prescription Dose 2340 cGy | | | | | | Prescription Dose 3500/3600 cGy | | | | | |
| --- | --- | --- | --- | --- | --- | --- | --- | --- | --- | --- | --- | --- | --- |
|  | Organ | HAL (cGy) | TB (cGy) | TBFF (cGy) | Difference (HAL-TB) (cGy) | HALFFF-TBFF (cGy) | TBFFF-TBFF (cGy) | HALFFF (cGy) | TBFFF(cGy) | TBFF (cGy) | Difference (HAL-TB)(cGy) | HALFFF-TBFF (cGy) | TBFFF-TBFF (cGy) |
| D0.2cc | Brainstem | 2454.9±38.3 | 2467.4±46.2 | 2444.8±29.7 | -12.5±45.7 | 621.4±1079.2 | 32.2±62.8 | 3718.8±97 | 3752.5±101.3 | 3788.6±88.7 | -33.7±77.9 | -69.8±77.1 | -36±66.7 |
|  | Cochlea (Bilateral) | 2346.1±81.3 | 2363.3±57.8 | 2370.3±40.8 | -17.1±59.4 | -24.2±91.5 | -7±73.3 | 3560.2±227.1 | 3577±136.4 | 3590.6±138.3 | -16.7±138.1 | -25.3±177.6 | -24.5±128.2 |
|  | Lens (Bilateral) | 565.7±238 | 561.8±200.8 | 470.4±203.9 | 3.9±110.8 | 125.9±259.7 | 129.1±220.4 | 860.9±438.9 | 880.0±418 | 922.8±404.8 | -18.1±101.5 | -61.9±107.6 | -42.8±98.8 |
|  | Optic Nerve (Bilateral) | 2307.3±152 | 2369.9±91.1 | 2355±128.3 | -62.6±148.2 | -47.7±213.7 | 14.8±136 | 3510.2±256 | 3390.8±326.7 | 3511.5±252.7 | 109.2±164.2 | -1.3±190 | -100.4±201.7 |
|  | Optic Chiasma (Bilateral) | 2401.8±68.3 | 2414.9±80.8 | 2378.4±38.6 | -12.4±60.3 | 23.4±84.1 | 39.9±85.5 | 3654.4±114.4 | 3662.9±84.8 | 3681.7±75 | -8.5±80.4 | -27.3±88.7 | -18.8±36.6 |
| Mean Dose | Eye (Bilateral) | 741.5±267.3 | 786.7±289.6 | 793.6±260.6 | -45.2±119.8 | -52.1±194.2 | -6.7±223.3 | 963.9±41.7 | 1002±451.1 | 1037.7±453.6 | -38.1±79.0 | -73.9±92.7 | -35.7±72.2 |
|  | Bladder | 93.4±94.6 | 107.8±95.7 | 120.6±97.1 | -14.3±20.9 | -27.1±38.6 | -12.8±45.9 | 170.4±165.1 | 192.5±164 | 191.1±115.9 | -22±31.9 | -20.6±132.2 | 1.4±124.1 |
|  | Duodenum | 757.5±335.2 | 766.5±316.5 | 742.8±494.1 | -9±39 | 14.7±372.5 | 23.7±352.2 | 1000.1±633.7 | 987.5±605.9 | 985.7±529.9 | 12.6±53.3 | 28.7±267 | 1.8±259.4 |
|  | Oesophagus | 1141.3±356.9 | 1223.5±412 | 1275.2±413 | -82.1±149.8 | -133.9±193.7 | -51.7±83.3 | 1357.2±294.8 | 1308.3±397.1 | 1442.3±279.6 | 48.9±190.6 | -85.1±69.7 | -134±184.4 |
|  | Femur Head (Bilateral) | 39±34 | 46.4±49 | 53.4±51.3 | -5.7±22 | -14.4±22.7 | -8.7±10.2 | 97.5±97 | 115.8±126 | 134±120.8 | -18.4±45.1 | -36.6±48.8 | -18.2±66.9 |
|  | Bowel | 483.6±143 | 538.7±164.5 | 593.1±172.7 | -55.1±35.4 | -109.5±117.2 | -54.4±104.8 | 705.6±162.8 | 770.4±183.6 | 768±221.6 | -64.8±48.3 | -59.6±56.3 | 2.1±41.7 |
|  | Heart | 432±148.9 | 474.5±188.6 | 331.1±216.6 | -42.5±92.4 | 100.9±241.4 | 143.4±284.4 | 547.2±140.4 | 560.2±179.8 | 531±232.6 | -13±66.1 | 7.1±258.5 | 29.3±273.3 |
|  | B/L Kidney | 418.1±172.1 | 470.1±207.4 | 454.6±184.1 | -52.0±95.8 | -36.6±166.1 | 15.4±136.4 | 524.9±114.1 | 540.4±126.3 | 630.4±126.5 | -15.4±59.6 | -105.4±176.5 | -89.9±193.8 |
|  | Larynx | 720.3±120.4 | 720.1±150.7 | 697.8±137.5 | 0.18±140.7 | 22.5±174.8 | 22.4±142.6 | 1041.6±209.3 | 961±188.2 | 929.95±193.1 | 80.6±178.8 | 111.6±221.3 | 31±100.7 |
|  | Liver | 398.4±92 | 426.9±107.6 | 443.2±106.5 | -28.6±43.7 | -44.9±72.2 | -16.3±73.8 | 569.5±174.5 | 607.3±180.1 | 477.9±106.1 | -37.7±33 | 91.6±180.6 | 129.4±189.6 |
|  | B/L LUNG | 482.6±155.1 | 541.9±153.8 | 549.3±133.7 | -59.3±65.3 | -66.7±74.7 | -7.4±50.3 | 751.0±270.1 | 822.0±274.3 | 821.4±260.1 | -70.9±84.2 | -70.4±105.7 | 0.6±86.9 |
|  | Mandible | 571.4±211.6 | 570.6±232.4 | 587.8±263.5 | 0.8±64.3 | -16.4±89.8 | -17.2±44.1 | 785.6±188.9 | 793.7±213.4 | 845.1±223.8 | -7.2±40.3 | -59.5±78.6 | -51.5±86.7 |
|  | Pancreas | 510.7±149.4 | 563.9±130.4 | 597.5±122.1 | -53.2±80.6 | -83.1±94.1 | -31±43.3 | 771.7±323.4 | 794.1±306 | 831.8±328.2 | -12.4±35.8 | -60.1±68.6 | -37.7±76.8 |
|  | B/L Parotid | 660.7±288.0 | 705.8±311.7 | 711.9±300.5 | -45.1±192.2 | -51.2±157.8 | -6±135.5 | 740.5±261.3 | 738.5±259.4 | 735.7±241.5 | 5.7±122.3 | 4.8±185.4 | 4.8±185.4 |
|  | Rectum | 165±125.5 | 197.6±155.5 | 234.7±179.2 | -32.6±41.6 | -69.7±217.6 | -37.1±232.3 | 374.3±201.7 | 429.6±229.5 | 452.5±241.2 | 55.3±72.4 | -78.2±93.2 | -22.9±56.1 |
|  | Gonads | 243.7±48.4 | 258.3±73 | 263.1±53.6 | -14.6±34.8 | -20.1±36.3 | -4.5±41.6 | 372.6±56.7 | 351.6±43.7 | 356.7±46.8 | 21±37.5 | 17.8±26.4 | -4.8±22.7 |
|  | Stomach | 407.8±148.2 | 451.4±131.8 | 532.5±180.2 | -43.6±105.8 | -124.7±295.8 | -81±274.3 | 624.0±242.7 | 640.5±221 | 669.7±198.1 | -16.4±51.9 | -45.4±60.7 | -29±45.7 |
|  | Thyroid | 907.9±332.6 | 912.6±404.1 | 863.7±392 | -4.7±102.3 | 44.2±238.5 | 48.9±252.2 | 1331.1±520 | 1295.2±575.9 | 1219.7±574.5 | 36±171.5 | 111.4±186.3 | 75.5±171.9 |

Supplementary table-1: comparative dose in cGy between three competing arms of the study (HAL6MVFFF, TB6MVFFF, and TB6MVFF), for two levels of prescription and their differences.
